# A comparison of patients’ and pharmacists’ satisfaction with medication counseling provided by community pharmacies: a cross-sectional survey

**DOI:** 10.1186/s12913-016-1374-x

**Published:** 2016-04-14

**Authors:** Seungwon Yang, Dasohm Kim, Hye Joung Choi, Min Jung Chang

**Affiliations:** Department of Pharmacy and Yonsei Institute of Pharmaceutical Sciences, College of Pharmacy, Yonsei University, 85 Songdogwahak-ro, Yeonsu-gu, Incheon 21983 Republic of Korea; Department of Pharmaceutical Medicines and Regulatory Science, Colleges of Medicine and Pharmacy, Yonsei University, Incheon, Republic of Korea

**Keywords:** Medication counseling, Satisfaction, Survey, Community pharmacists, Patients

## Abstract

**Background:**

Medication counseling is a critical component of pharmaceutical care to promote the safe and effective use of medications and to maximize therapeutic outcomes. The assessment of patients’ and pharmacists’ satisfaction with medication counseling services could be one of the vital parameters for predicting the quality of pharmacy services. No study has measured and compared both patients’ and pharmacists’ satisfaction with medication counseling. The objectives of this study were to describe and compare patients’ and pharmacists’ levels of satisfaction with medication counseling services offered by community pharmacists in South Korea.

**Methods:**

This was a descriptive, cross-sectional survey. The online survey was distributed to patients and community pharmacists using a structured questionnaire. The questionnaires consisted of 4 main areas: (1) responders’ characteristics (2) current state of medication counseling methods provided by community pharmacies (3) overall satisfaction with medication counseling (4) demand for the development of medication counseling standards. A comparison between patients and pharmacists was made using either a chi-square test or a Fisher’s exact test.

**Results:**

Between June 13, 2014 and July 15, 2014, a total of 252 patients and 620 pharmacists completed the survey. It was found that 47.3 % of pharmacists and 34.0 % of patients were satisfied with the current medication counseling service. Pharmacists showed a higher degree of satisfaction with the medication counseling service compared to patients (*p* <0.05). A major reason for patients not being satisfied with the medication counseling from community pharmacists was the insufficient time spent on counseling (51.2 %). The pharmacists’ perception of a major barrier to providing appropriate medication counseling for patients was the lack of time (24.3 %). Moreover, a substantial number of patients (88 %) and pharmacists (73 %) supported the development of medication counseling standards to improve community pharmacist counseling services (*p* < 0.001).

**Conclusions:**

This study showed that both patients and pharmacists have low levels of satisfaction with the current medication counseling service offered by community pharmacists. This study provides baseline data for the development of national guidelines for medication counseling by pharmacists.

## Background

Medication counseling has become a key priority for modern community pharmacists. Modern pharmacy practices display an evolving paradigm from traditional drug dispensing to more active and expanded clinical roles, including patient-oriented medication counseling activities [1, 2]. Medication counseling refers to “providing medication information orally or in written form to the patients or their representatives on directions of use, advice on side effects, precautions, storage, diet, and lifstyle modifications”[3]. Preliminary studies showed that through medication counseling, pharmacists may identify and correct drug-related problems, improve the patient’s knowledge about the proper use of medicines, increase patient satisfaction with the pharmacy service, and consequently optimize the patient quality of care [4–6].

Satisfaction assessment is considered an important indicator of the quality of the pharmacy service as it reflects whether the service is meeting one’s expectations or values. There is an increasing trend to assess satisfaction level when the pharmacy service has started to expand its scope of practice [7–10]. So far, previous studies have focused on the patients’ or pharmacists’ satisfaction with pharmacy services or with specific disease management services in developed countries, which is often difficult to generalize to other countries, such as South Korea, where the pharmacy service is still more likely to use the traditional role. Medication counseling practice by community pharmacists has been studied by performing surveys and evaluating patients’ or pharmacists’ satisfaction [9, 10]. Evaluating the level of satisfaction with medication counseling has become one of the pivotal components for predicting the quality of pharmacy services [10].

In South Korea, much attention has recently been focused on the issue of medication counseling practice by community pharmacists. Although it has been legally mandated that pharmacists are required to provide medication counseling to every patient [11], pharmacists have often failed to offer drug information to patients or only provide brief counseling upon patient request. As a result, more-stringent regulations were recently announced imposing monetary penalties on pharmacists who do not provide medication information to patients with each prescription filled [12]. However, this mandate has not necessarily worked as intended, and the quality and content of information provided varies among pharmacists [13]. Although this clearly calls for the quality assessment of medication counseling at this point, no studies have been conducted to evaluate the satisfaction with medication counseling by community pharmacists. To date, no studies have compared pharmacists’ and patients’ view or satisfaction with the medication counseling practice. The feedback from a survey could help to identify differences in levels of satisfaction with medication counseling between pharmacists and patients, and could assist in pinpointing areas for future improvement.

This study represents the first attempt to measure and compare patients’ and pharmacists’ satisfaction with medication counseling services provided by community pharmacists in South Korea. To evaluate the quality of current medication counseling practice by community pharmacists, questionnaires are an effective means for obtaining feedback on patients’ or pharmacists’ counseling experiences. In addition, the findings of this study will provide baseline data and strong evidence for improving the quality of medication counseling by community pharmacists.

One aim of this study was to assess and compare the levels of satisfaction of patients and community pharmacists with current medication counseling. We also aimed to examine the expectations of both patients and pharmacists regarding the implementation of medication counseling standards as a strategy to improve the quality of medication counseling.

## Methods

A descriptive, cross-sectional survey was designed to explore pharmacists’ and patients’ levels of satisfaction with the current provision of medication counseling services provided by community pharmacists in South Korea from June 13, 2014 to July 15, 2014. The research was approved by the Yonsei University Institutional Review Board (IRB no. 1040917-201405-SB-170-02) and conducted in accordance with the Declaration of Helsinki. Informed consent was obtained from all participants before the administration of the survey. Participation in this survey was voluntary, and confidentiality was maintained throughout the study.

### Study population

The pharmacist survey was intended for pharmacists who were currently practicing in community pharmacies in South Korea at the time of the study. The patient survey was intended for adults who had received medication counseling from community pharmacists. A convenience sampling method was employed in this survey to facilitate the recruitment of a large sample and to attract respondents from large geographical areas.

### Questionnaire development

Two instruments were developed to measure the satisfaction level of patients and community pharmacists with the medication counseling service. Each self-administered questionnaire was developed primarily from published literature on medication counseling and expert reviews. Prior to data collection, the contents of the questionnaires were reviewed by groups of pharmacy researchers and experts for relevance, appropriateness, and acceptability. The face validity of the questionnaire was assessed by groups of practicing community pharmacists and pharmacy-users for clarity and comprehension. Then, the questionnaire was pretested with 25 patients and 10 community pharmacists and adjusted accordingly (the pilot sample was not included in the study sample). Reliability assessment was computed for the satisfaction responses to ascertain the internal consistency of the questionnaires using Cronbach’s alpha, which yielded 0.86 for the pharmacist survey and 0.65 for the patient survey.

The questionnaires for the patient and pharmacist surveys included four main domains:

1. Respondents’ characteristics
2. Current state of medication counseling methods provided by community pharmacies
  Patient questionnaire: perceived time taken for medication counseling, counseling method, and source of drug information obtained
  Pharmacist questionnaire: perceived time spent on counseling and counseling method
3. Overall satisfaction with medication counseling
  Patient questionnaire: overall satisfaction and reasons for dissatisfaction with medication counseling
  Pharmacist questionnaire: overall satisfaction, barriers to counseling patients, and reasons for dissatisfaction with medication counseling
4. Demand for the development of medication counseling standards

Both the patient and pharmacist questionnaires consisted of 5-point Likert scales with descriptive choices ranging from “very satisfied” to “very dissatisfied” to measure and compare the satisfaction level between patients and pharmacists, and choices ranging from “very necessary” to “very unnecessary” to assess the demand for the development of medication counseling standards. Moreover, a variety of open- and close-ended questions was included in the questionnaires with a section to write comments.

### Questionnaire distribution

A web-based survey was undertaken by both community pharmacists and patients. Both questionnaires were distributed online using the Qualtrics software, which allows respondents to navigate easily through a series of questions [14]. For the patient survey, an online questionnaire was posted on social network services, such as Facebook, Twitter, and so forth, using a hyperlink. For the pharmacist survey, the survey link was distributed through Pharm Manager 2000 (Korea Pharmaceutical Information Centre, Seoul, Korea), which is the pharmacy management software most widely utilized in community pharmacies in South Korea. On Qualtrics, a tool was enabled to preclude respondents from performing multiple entries using the same IP address and device while maintaining the anonymity of respondents.

### Data handling and statistical analyses

Once the survey was closed, data were downloaded from the Qualtrics website and imported directly into SPSS version 18.0 (SPSS Inc., Chicago, IL, USA) for analysis. The accuracy of the gathered data was assessed by visual inspection by two researchers who were independent of the study. During the assessment, the researchers eliminated inconsistent and unreliable answers (e.g., contradictory answers or all the same answers for a series of questions) from the final analysis. Descriptive statistics were calculated to present the frequencies of the survey responses. A comparison between patients and pharmacists was made using either a Chi-square test or a Fisher’s exact test. Differences between the groups were considered statistically significant if the p-value was less than 0.05.

## Results

### Survey participants

**Fig. 1:**
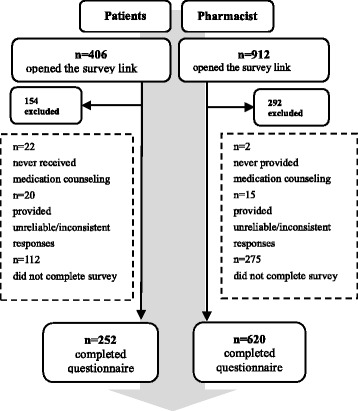
Flow chart showing selection of the study participants

Between June 13, 2014 and July 15, 2014, a total of 252 patients and 620 pharmacists participated in this survey. For the patient survey, of the 406 patients who opened the survey link, 252 patients (62.1 %) completed the questionnaire (response rate: 62.1 %). One hundred and fifty-four (37.9 %) patients were excluded because 22 had never received medication counseling from community pharmacists, 20 gave inconsistent and unreliable responses (e.g., contradictory answers or all the same answers for a series of questions), and 112 did not complete the questionnaire. Thus, 252 of 406 patient surveys were included in the final analysis. For the pharmacist survey, of the 912 pharmacists who opened the survey link posted on Pharm Manager 2000, 620 (67.9 %) completed the questionnaire (response rate: 68.0 %). Two hundred and ninety-two (32.0 %) pharmacist questionnaires were excluded from the final analysis because two had never performed counseling services, 15 gave inconsistent and unreliable responses, and 275 did not complete the questionnaire (Fig. 1).

The characteristics of the survey respondents are shown in Table 1. For the patient survey, most patients were younger than 40 years (64.3 %) and female (67.1 %). For the pharmacist survey, approximately half of the pharmacists was male (52.7 %), aged over 50 years (51.3 %), and had more than 20 years (50.5 %) of professional experience. Both patients and pharmacists were from all regions of South Korea.

**Table 1:** Characteristics of the patients and pharmacists

	Number of patients (%) total = 252	Number of pharmacists (%) total = 620
Age (years)		
20–29	69 (27.4)	22 (3.5)
30–39	93 (36.9)	109 (17.6)
40–49	45 (17.9)	171 (27.6)
50–59	15 (5.9)	200 (32.3)
60 or older	30 (11.9)	118 (19.0)
Gender		
Male	83 (32.9)	327 (52.7)
Female	169 (67.1)	293 (47.3)
Geographic region^a^		
Seoul	80 (31.7)	131 (21.1)
Gyeonggi-do/Incheon	52 (20.6)	150 (24.2)
Chungcheong-do	34 (13.5)	64 (10.3)
Gyeongsang–do	49 (19.4)	185 (29.8)
Jeolla-do	34 (13.5)	17 (2.8)
Gangwon-do	1 (0.4)	64 (10.3)
Jeju Island	2 (0.8)	9 (1.5)
Years of practice		
Less than 1 year	-	4 (0.6)
1–4 year(s)	-	37 (6.0)
5–9 years	-	67 (10.8)
10–14 years	-	101 (16.3)
15–19 years	-	98 (15.8)
20 or more years	-	313 (50.5)

^a^Pharmacists: geographic region of practice

### Current state of medication counseling methods

**Fig. 2:**
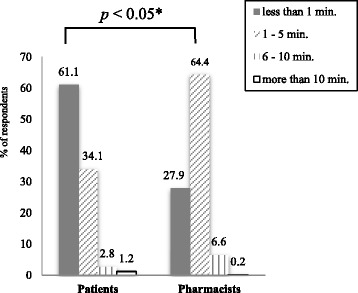
A comparison of perceived time taken for medication counselling between patients and pharmacists. * *p* < 0.05, chi-square test

With respect to the patients’ responses to the time taken for medication counseling, 61.1 % of patients perceived the length of time to be < 1 min, 34.1 % perceived it to be 1–5 min, and 4.0 % perceived it to be > 5 min. The pharmacists reported the length of time they spent on medication counseling per patient to be 1–5 min (64 %), < 1 min (27.9 %), and > 5 min (6.8 %), as shown in Fig. 2. The perceived time taken of patients and pharmacists was significantly different (*p* < 0.05)

The most common method of medication counseling reported by both patients and pharmacists was verbal counseling (90.4 % vs. 96.2 %, *p* < 0.001), followed by medication information printed on the prescription bag (*p* = 0.142), additional printout or stickers (*p* < 0.001), physical demonstration (*p* < 0.001), mobile application (*p* < 0.001), or visual and auditory materials (*p* = 0.070) (Table 2).

**Table 2:** Current state of medication counseling methods provided by community pharmacies

Variables	Number of Patients (%) total = 252	Number of Pharmacists (%) total = 620	*p-*value*
Counseling methods^a^			
Verbal	229 (90.4)	599 (96.2)	< 0.001
Medication information printed on the prescription bag	139 (54.8)	308 (49.4)	0.142
Additional printout or stickers	18 (7.1)	155 (24.8)	< 0.001
Physical demonstration	7 (2.8)	91 (14.6)	< 0.001
Mobile application	0 (0)	73 (11.8)	< 0.001
Visual and auditory materials	1 (0.4)	13 (2.0)	0.070
Others^b^	1 (0.4)	22 (3.5)	0.008
Missing data	2 (0.8)	6 (1.0)	
Total	465 (156.7)	1261 (203.3)	
Sources of drug information obtained			
Portal site search (e.g., Google)	130 (51.6)	-	
Inquiry to pharmacists	69 (27.4)	-	
Inquiry to doctors	27 (10.7)	-	
Medical website	22 (8.7)	-	
Government website (e.g., Ministry of Food and Drug Safety)	2 (0.8)	-	
Others^c^	1 (0.4)	-	
Missing data	1 (0.4)	-	
Total	252 (100.0)	-	

*Chi-square test

^a^Total percentage may exceed 100 % as participants were asked to give multiple responses

^b^Example: medication calendar, labeling, etc.

^c^Example: package inserts

When patients had any medication-related questions, they reported that portal sites (Naver, Daum, and Google, etc.) were the most frequent source of drug information (51.6 %), followed by pharmacists (27.4 %), physicians (10.7 %), medical websites (8.7 %), and government websites (0.8 %).

### Overall satisfaction with medication counseling

**Fig. 3:**
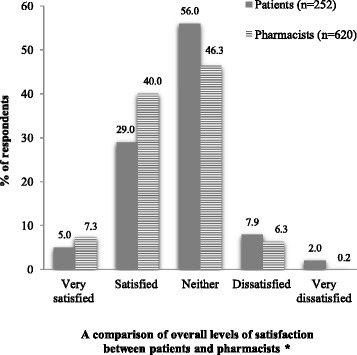
A comparison of overall level of satisfaction with medication counseling between patients and pharmacists. * *p* <0.05, Fisher’s exact test

**Fig. 4:**
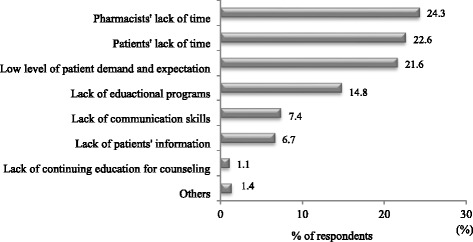
Community pharmacists’ responses of perceived barriers to medication counseling. Note: Others included lack of reimbursement for service, decreased revenue, lack of legal support, etc.

Only 34.0 % of patients were “satisfied” (29.0 %) or “very satisfied” (5.0 %) with the current medication counseling service provided by community pharmacists. Of the pharmacists, 47.3 % were “satisfied” (40.0 %) or “very satisfied” (7.3 %) with the medication counseling practice they provide for patients. There were significant differences between the two groups in that pharmacists showed a higher degree of satisfaction with the counseling service compared to patients (*p* < 0.05) (Fig. 3). The top three reasons for patients being dissatisfied with medication counseling included “insufficient time for counseling” (51.2 %), “use of supplementary counseling aids” (36.0 %), and “privacy” (11.1 %) (Table 3). For the pharmacist survey, the three main barriers to patient counseling included “pharmacists’ lack of time” (24.3 %), “patients’ lack of time” (22.6 %), and “low levels of patient demand and expectation” (21.6 %) (Fig. 4). Other barriers are described in Fig. 4.

**Table 3:** Patients’ reasons for dissatisfaction with medication counseling

Reasons	Number of patients (%)
Pharmacists’ attitudes	17 (6.8)
Use of plain language	16 (6.3)
Content of medication information received	36 (14.3)
Insufficient counseling time	129 (51.2)
Use of supplementary counseling aids	91 (36.0)
Privacy	28 (11.1)
Total	317 (125.7)

Total percentage may exceed 100 % as participants were asked to give multiple responses

**Fig. 5:**
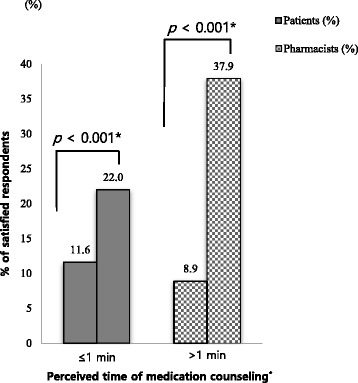
A comparison of satisfaction level according to perceived time between patients and pharmacists. * chi-square test

The satisfaction level was further assessed based on the perceived time taken for medication counseling (Fig. 5). Patients who received more than 1 min of medication counseling were significantly more satisfied than those who received less than 1 min (*p* < 0.001). Pharmacists who spent more than 1 min on medication counseling were approximately four times more satisfied with their medication counseling practice (*p* < 0.001).

### Demand for the development of medication counseling standards

Although the majority of patients and pharmacists supported the development of medication counseling standards, there were significant differences between patients and pharmacists (*p* < 0.001). The majority of patients responded that it was “very necessary” (34.9 %) or “necessary” (52.8 %) to develop medication counseling standards. Over two-thirds of the pharmacists responded that it was “very necessary” (30.0 %) or “necessary” (43.1 %) to develop medication counseling standards (Table 4).

**Table 4:** Patients’ and pharmacists’ demand for the development of medication counseling standards

	Number of participants (%)		*p-*value*
	Patients	Pharmacists	
Very necessary	88 (34.9)	186 (30.0)	< 0.001
Necessary	133 (52.8)	267 (43.1)	
Neither	26 (10.3)	105 (16.9)	
Unnecessary	3 (1.2)	45 (7.3)	
Very unnecessary	2 (0.8)	17 (2.7)	
Total	252 (100.0)	620 (100.0)	

*Chi-square test

## Discussion

This is the first study, to our knowledge, that has assessed and compared patients’ and pharmacists’ levels of satisfaction with medication counseling services performed by community pharmacists in South Korea. The results of this study showed that the current medication counseling service was associated with low levels of both patient and pharmacist satisfactions. More than half of the respondents in both the patient and pharmacist groups were not satisfied with the medication counseling service and patients appeared to have less favorable views of the counseling service received from community pharmacists.

The results of this study are slightly inconsistent with a previous study that evaluated the quality of current medication counseling in community pharmacies. Shin et al. [13] reported that the pharmacists’ satisfaction with their own medication counseling was relatively less favorable (31.1 %) compared to the present study (47.3 %) [13]. The higher degree of pharmacists’ satisfaction in the present study may be due to the use of a different method and the different survey content to the previous study. Another previous study, by Van Geffen et al. [15], evaluated patient satisfaction with counseling by pharmacists [15]. The level of patient satisfaction with counseling was inconsistent with that found in the present study: patient satisfaction was slightly higher in the previous study compared to the present study (42.0 % vs. 31.1 %). This inconsistency could be due to the different study setting and the selection of the patient population. The patients’ or pharmacists’ satisfaction has a practical implication for evaluating the quality of pharmacy service [16]. An overall low level of satisfaction with the medication counseling service in both groups indicates that the quality of medication counseling does not meet the patients’ needs and expectations. Counseling should be more tailored to the patients’ needs. Pharmacists need to expand their roles in supporting patients with their medications.

The present study investigated the reasons why patients were not satisfied with medication counseling received from community pharmacists. The patients pinpointed the length of counseling time as a main reason for dissatisfaction with the medication counseling service. Interestingly, pharmacists also perceived time constraints as a major barrier to delivering effective medication counseling practice, which was similar to previous studies [13, 17, 18]. The role of pharmacists is dominated by dispensing and checking the high volume of prescriptions in South Korea. With the emphasis on the pharmacist’s responsibility for medication counseling, pharmacists have an increased workload and increased job stress levels, which may contribute to the poor performance of pharmacists as well as the poor quality of the pharmacy service [19]. Effective solutions for alleviating the pharmacists’ time constraint issue include the improvement of pharmacy staffing levels and optimal time management [19, 20]. The pharmacy staffing levels can be improved by adequately reimbursing pharmacists for the time spent on medication counseling [21]. Additionally, it is important for pharmacists to plan strategies for time management to overcome job stress and to aid the accomplishment of their responsibilities more efficiently. Optimal time management will allow pharmacists to spend more time interacting and communicating with the patient, thereby improving the quality of the pharmacy service. The majority of pharmacists perceived that they spent 1–5 min counseling patients, whereas the majority of patients reported that the length of time taken for medication counseling was less than 1 min. As the length of time taken for counseling increased, the levels of patient and pharmacist satisfaction significantly increased, which may be related to an increased amount of relevant information imparted to patients with increased counseling time. The provision of better levels of information has been associated with an increased satisfaction level [22, 23]. Thus, pharmacists should attempt to allocate sufficient time to deliver effective medication counseling to patients.

It is important for pharmacists to provide appropriate, clear, and relevant information to patients about their medications. Given the advertising of medications in the media and easy access to medical information on websites, patients were most likely to rely on portal sites first to search for medication information before they asked pharmacists, as shown in the present study. Even though the patients’ satisfaction with the pharmacists’ performance of medication counseling was low in the present study, the pharmacists were still considered a reliable source for patients when seeking medication information. Pharmacists are in a unique and readily available position to answer patients’ concerns and inquiries about their medications that they may read about or hear about from others.

The present study also suggested that national guidelines for medication counseling standards or protocols should be implemented and developed for effective medication counseling in addition to verbal counseling. Even though a new pharmaceutical affair law reinforces pharmacist counseling practices, the contents of medication information that pharmacists should provide to patients was lacking and often varied from pharmacist to pharmacist. This clearly calls for the contents of medication information to be standardized in a more patient-oriented direction or as user-tailored content. Based on the medication counseling standards, pharmacists could use their expertise in determining which information should be provided to each patient, with the expectation of improving the quality of medication counseling. The U.S. Food and Drug Administration has already published *Medication Guides*, which are paper handouts that contain information for patients on how to use a medication safely [24]. The guides are distributed with many prescription drugs that pharmacists dispense to patients. As shown in previous studies, providing structured and standardized written information in addition to verbal counseling can help patients or their caregivers to better understand their medications [25, 26].

## Conclusions

The findings of this study should be interpreted with some limitations. There is potential bias associated with the convenience sampling method, which may reduce the generalizability of this study. However, convenience sampling was employed in the present study in order to recruit as many respondents as possible from a wide geographical area. Another limitation is the questionnaire distribution method used in this study. As this survey was carried out via the Internet, the access to the survey may have been limited for elderly people who may have a different experience and expectation of the service. Patients older than 50 years were under-represented in this study, which may have been due to selection bias. Moreover, the responses of patients and pharmacists relied on their ability to recall information and experiences of medication counseling practices, which may have resulted in recall bias.

Despite these limitations, this survey has provided useful insight into the current state of medication counseling practices and satisfaction levels of both patients and pharmacists. The low satisfaction levels of patients and pharmacists may reflect the low quality of medication counseling currently provided in community pharmacies in South Korea. Both the patient and pharmacist groups greatly supported the implementation of a national level of medication counseling standards as one of the strategies to improve the quality of pharmacy services. Consequently, this study provides baseline data for the construction of strategies to improve pharmacist medication counseling services in South Korea.

### Ethics approval and consent to participate

The research was approved by the Yonsei University Institutional Review Board (IRB no. 1040917-201405-SB-170-02) and conducted in accordance with the Declaration of Helsinki. Informed consent was obtained from all participants before the administration of the survey. Participation in this survey was voluntary, and confidentiality was maintained throughout the study.

### Availability of data and materials

The datasets supporting the conclusions of this article are presented and included in the results section of the main manuscript.
